# Significance of seed dispersal by the largest frugivore for large-diaspore trees

**DOI:** 10.1038/s41598-022-23018-x

**Published:** 2022-11-21

**Authors:** Hiroki Sato

**Affiliations:** Graduate School of Asian and African Area Studies, Kyoto University, 46 Shimoadachi-Cho, Yoshida, Sakyo-Ku, Kyoto, 606-8501 Japan

**Keywords:** Ecology, Plant sciences

## Abstract

How do large-bodied frugivores contribute to seed dispersal of large-diaspore plants? This study examined seed dispersal effectiveness for two large-diaspore tree species, *Astrotrichilia asterotricha* (*AA*) and *Abrahamia deflexa* (*AD*), in a Madagascan forest. I evaluated fruit removal rates through focal tree observations and factors affecting seedling recruitment up to the 2-year-old seedling stage. I confirmed brown lemur (*Eulemur fulvus*) as the sole disperser, removing 58.8% and 26.0% of fruits produced by *AA* and *AD*. Brown lemurs frequently visited large-crowned *AA* trees with high density of fallen fruits and more adjacent fruiting trees during seasons with low fruit diversity. Most *AA* seedlings were removed by predators, although canopy openness slightly improved seedling establishment. Although *AD* seeds were severely attacked by predators under mother trees, the seedlings survived under dispersal conditions distant from the mother trees, and with low density of diaspores. *AD* had a higher cumulative probability from fruit removal to seedling recruitment (6.5%) than *AA* (1.5%) in the first rainy season. This study clarifies the significance of seed dispersal to tree recruitment strategies, which vary among different combinations of tree species and large frugivores, i.e. quantitative dispersal to reach suitable microhabitats, and qualitative dispersal to escape from dangerous zones near mother trees.

## Introduction

Seed dispersal represents a plant strategy to enhance reproductive success, and recruitment patterns presumably influence population dynamics and biodiversity at the community level^1, 2^. Seed dispersal by animals has been studied in detail in tropical forests, because 50–90% of plant species in these ecosystems are endozoochorous^1, 3^. The roles of seed dispersal agents have been evaluated systematically in accordance with the concept of seed disperser effectiveness (SDE), expressed as the product of quantitative and qualitative components, i.e. the number of seeds dispersed from fruiting mother plants and the probability of seed/seedling survival and recruitment as new adults, respectively^4^. The SDE has mostly been evaluated separately for quantitative component in the dispersal phase from fruit production to seed dispersal and qualitative component in the post-dispersal phase from seed deposition to new recruitment as surviving seedlings^2, 5, 6^. However, the definition of SDE has been broadened to “seed dispersal effectiveness”, emphasizing the need to evaluate the overall effectiveness of the full process of plant regeneration from seed dispersal to seedling establishment and survival^7^. As such, recent studies have comprehensively evaluated SDE by incorporating both the quantitative and qualitative components^8–12^.

During the dispersal phase, the quantitative component of SDE has mainly been evaluated by observing fruit removal from focal plants by animals^13–15^. To gain an understanding of the mechanisms of successful fruit removal, researchers have examined intrinsic (plant size, fruit density, pulp ratio per fruit, and so forth) and extrinsic factors (fruiting of neighbouring trees, fruit availability of the forest, and so forth)^16^. Examination of the feeding strategies of frugivores has indicated that some birds visit trees with high pulp ratio per fruit^14^, and that primates and birds remove more fruits from trees with larger crop sizes^14, 17–19^. As the main component of quantitative effectiveness, the relative quantity of fruit removal has been mostly evaluated by comparisons among seed disperser assemblages^15, 20^. However, to evaluate dispersal effectiveness rather than disperser effectiveness, it is necessary to evaluate the fruit removal rate of each targeted animal taxon in the amount of fruit production as an absolute quantitative assessment of SDE in the dispersal phase of focal plants^13, 14, 16^.

During the post-dispersal phase, the probability of seedling recruitment, the primary qualitative component of SDE, has been evaluated by monitoring the fates of seeds or seedlings, which is influenced by predation, secondary dispersal, pathogen disease, and stress among other environmental factors^21–24^. Such monitoring has indicated high mortality rates of seeds/seedlings at sites with high seed densities and close to conspecific fruiting trees, known as the Janzen-Connell effect^23, 25–28^. Monitoring studies have also reported high survival rates and/or high growth rates in open-canopy microhabitats^21, 23^ and high survival rates via secondary dispersal^24, 29, 30^. Some studies that have evaluated SDE by multiplying the quantitative and qualitative components report that the initial effects of fruit removal by frugivores during the dispersal phase are often hindered by high and heterogeneous mortality^13, 31–33^, whereas others have detected effects of frugivores on seedling recruitment patterns^34, 35^. Thus, further study is required for a more holistic understanding of SDE.

In recent years, low densities of seedlings/saplings of large-seeded plants and aggregated distribution around mother trees were found in empty and half-empty forests following the disappearance or decrease in number of large frugivores^36–39^. These findings suggest that in species-depauperate networks formed by strong mutualism between large-seeded plants and large-bodied frugivores, a small number of key frugivores have a large SDE and provide robust connectivity between the dispersal and post-dispersal phases. This hypothesis was recently tested by evaluating the SDE of primates, bears, and elephants for large-seeded plants growing in a seasonal evergreen forest in Thailand^8, 10, 12^. However, such studies have only reported on a limited number of taxonomic groups and involved few study sites. Furthermore, since the concept of attracting and introducing frugivores as effective restoration agents for reforestation has been proposed^40–42^, to understand SDE of large frugivores is also an important issue in conservation biology.

**Figure 1:**
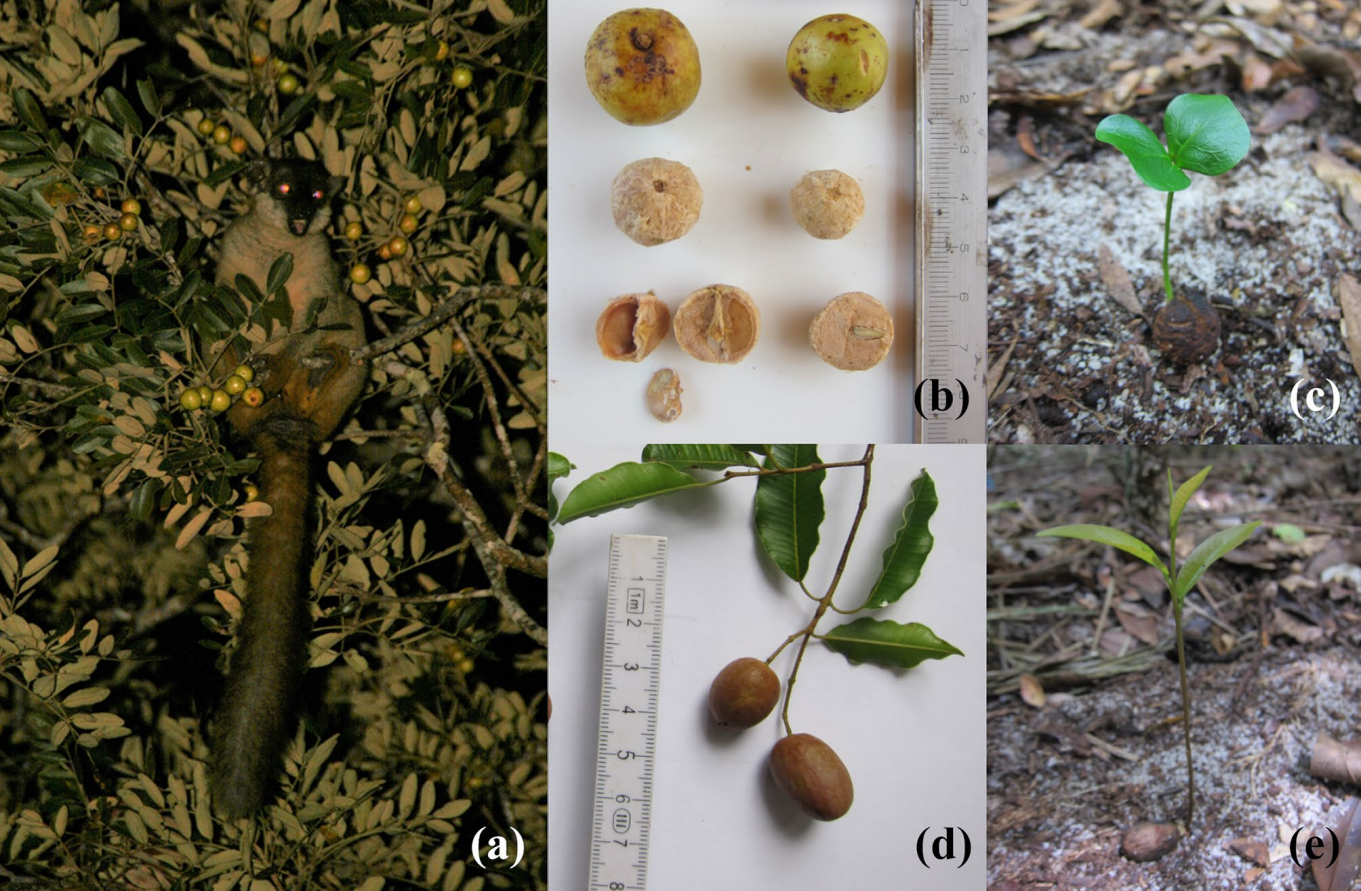
Study subjects. (**a**) The common brown lemur (*Eulemur fulvus*) feeding in a fruiting canopy of *Astrotrichilia asterotricha*. (**b**) Fruits covered with a hard shell and small seeds of *Astrotrichilia asterotricha*. (**c**) Seedling of *Astrotrichilia asterotricha* with phanerocotylar epigeal foliaceous cotyledons. (**d**) Fruits of *Abrahamia deflexa*. (**e**) Cryptocotylar hypogeal seedling of *Abrahamia deflexa* with reserve-storage cotyledons.

To examine the SDE of large frugivores for large-seeded plants, this study focused on seed dispersal systems in Madagascar, where the frugivorous guild is depauperate of large avian and mammalian seed dispersers^43, 44^. In Madagascan forest ecosystems, the largest frugivorous taxon, Lemuridae (body mass: 1.6–3.6 kg^45^), has proven to be a highly effective seed disperser, i.e. they are capable of dispersing large quantities of intact seeds from a variety of plant species over large areas^46–50^. In addition, they are thought to be the sole seed dispersers for large-seeded plants^43, 45, 48^. The total loss or reduction of Lemuridae has resulted in decreased regeneration success among large-seeded plants^51^ and may cause a decrease in the above-ground carbon storage of forests^52^. However, the SDEs of Lemuridae for large-seeded plants with links from fruit production to seedling recruitment have rarely been examined^53^. In a dry deciduous forest of north-western Madagascar, the largest frugivore, the common brown lemur [*Eulemur fulvus* (Fig. 1), body mass = 1.6–2.4 kg (N = 11), Sato, unpublished data], disperses seeds of 70 plant species, 23 of which depend on this primate for seed dispersal due to the large size of their diaspores (> 10 mm in diameter)^48^. As the inter-specific difference in dispersal and post-dispersal phases can be critically important for understanding distinctive patterns of initial tree recruitment^54, 55^, this study examined the SDEs of the brown lemur for two large-diaspore tree species. *Astrotrichilia asterotricha* (Radlk.) Cheek (Meliaceae; hereafter *AA*) and *Abrahamia deflexa* (H. Perrier) Randrian and Lowry (Anacardiaceae; hereafter *AD*) comprise the major sources of fruit in the diet of the lemurs during the dry and rainy seasons, respectively (Fig. 1)^48, 56^. That these two tree species occur at moderate densities in the forest (see "Methods") may be an indication of effective population regeneration due to the high SDE of brown lemurs.

The objective of this study was to understand the significance of seed dispersal by this large frugivore in the seed dispersal and recruitment strategies of large-diaspore trees by linking the fruit removal and seedling establishment stages. To assess the expected high SDE of brown lemurs, I addressed the following questions: how many fruits do brown lemurs remove from focal trees, and what tree traits increase the visit frequency by seed dispersers?; do seed dispersal conditions (escape from fruiting trees) improve the probability of seedling recruitment, and what factors increase recruitment success?; and what is the significance of seed dispersal by brown lemurs, as inferred by multiplying the quantitative and qualitative components of the SDE?

## Results

### Effective seed dispersers in visitor assemblages

A total of 4 mammal species and 10 bird species visited the crowns of *AA* or the forest floor below the crown, and the total time that these animals spent in *AA* was 55 h 48 min 19 s (out of 360 h of focal tree observations) (Supplementary Table S1 online). Of these visiting animal species, those that foraged for *AA* fruits were limited to brown lemurs and Milne-Edwards’ sportive lemurs (*Lepilemur edwardsi*; see Supplementary Table S1 online for detailed behaviour of each species at *AA* trees). Feeding behaviour on *AA* fruits by brown lemurs occupied 69.6% of the time, and they mostly swallowed the fruits. Although I also recorded fruit consumption by sportive lemurs (1.7% of the total time spent by animals), they never swallowed the fruits, but rather spat them out beneath the crown of the focal tree.

Six mammal species and 7 bird species visited the crowns of *AD* trees or the forest floor below the crowns, and the total time spent by all visitors was 7 h 42 min 27 s (out of 280 h of focal tree observations) (Supplementary Table S2 online). Of these animals, only two species, brown lemurs and fat-tailed dwarf lemurs (*Cheirogaleus medius*), foraged for *AD* fruits (see Supplementary Table S2 online). Feeding behaviour on *AD* fruits by brown lemurs occupied 39.6% of the time and they typically swallowed the fruits. Although dwarf lemurs also spent considerable time feeding on *AD* fruits (30.9%), they spat the seeds out under the crowns.

### Effects of tree traits on the frequency of brown lemur visits

The proxy for the frequency of brown lemur visits (i.e. the frequency of finding brown lemur faeces in the fruit traps every 2 days during the fruiting period) and variations in traits among focal trees are summarised in Table 1. Variables affecting tree selection by brown lemurs were assessed using generalised linear models (GLMs) with a Poisson distribution and log-link function. The proxy of visit frequency was the dependent variable, while the independent variables included crown area, the density of fallen fruits, the fresh weight of pulp per fruit, and the number of adjacent fruiting trees (see "Methods" for details). Visit frequency was highly variable among focal *AA* trees, and brown lemurs frequently visited fruiting trees with larger crown areas, higher densities of fallen fruits, and more adjacent fruiting trees (Table 2). With respect to *AD*, brown lemurs more frequently visited trees with higher densities of fallen fruits (Table 2).

**Table 1:** The proxy of visit frequency by brown lemurs and the variation in tree traits in focal trees of *Astrotrichilia asterotricha* and *Abrahamia deflexa*.

Species	Astrotrichilia asterotricha		Abrahamia deflexa	
Variable	Mean + SD	Range	Mean + SD	Range
Proxy of visit frequency (N)	6.9 ± 6.9	0–20	4.3 ± 2.3	1–9
Crown area (m^2^)	26.2 ± 16.0	10.0–66.5	31.0 ± 15.1	7.1–58.8
Density of fallen fruits (N/m^2^)	26.9 ± 18.0	5.3–78.0	31.3 ± 20.8	10.7–72.0
Pulp weight / fruit (g)	1.00 ± 0.14	1.30–0.69	0.57 ± 0.06	0.68–0.48
Neighbor fruiting trees (N)	6.5 ± 3.6	1–15	4.2 ± 2.3	0–7

**Table 2:** Results of generalized linear model analyses for the effects of tree traits on the proxy of visit frequency by brown lemurs in focal trees of *Astrotrichilia asterotricha* and *Abrahamia deflexa*.

Species	*Astrotrichilia asterotricha*		*Abrahamia deflexa*	
Independent variable	Coefficient ± SE	*P* value	Coefficient ± SE	*P* value
Intercept	− 4.13 ± 1.07	0.0001	1.87 ± 2.03	0.36
Crown area (m^2^)	0.068 ± 0.012	< 0.0001	0.021 ± 0.017	0.20
Density of fallen fruits (N/m^2^)	0.017 ± 0.0060	0.014	0.016 ± 0.0067	0.017
Pulp weight / fruit (g)	− 1.81 ± 1.19	0.13	− 2.58 ± 4.06	0.52
Neighbor fruiting trees (N)	0.17 ± 0.050	0.0007	− 0.054 ± 0.069	0.43
AIC (AIC of null model)	63.3 (144.3)		54.0 (60.5)	
Residual deviance	18.1 on 8 degrees of freedom		2.85 on 8 degrees of freedom	

### Fruit removal by brown lemurs

**Figure 2:**
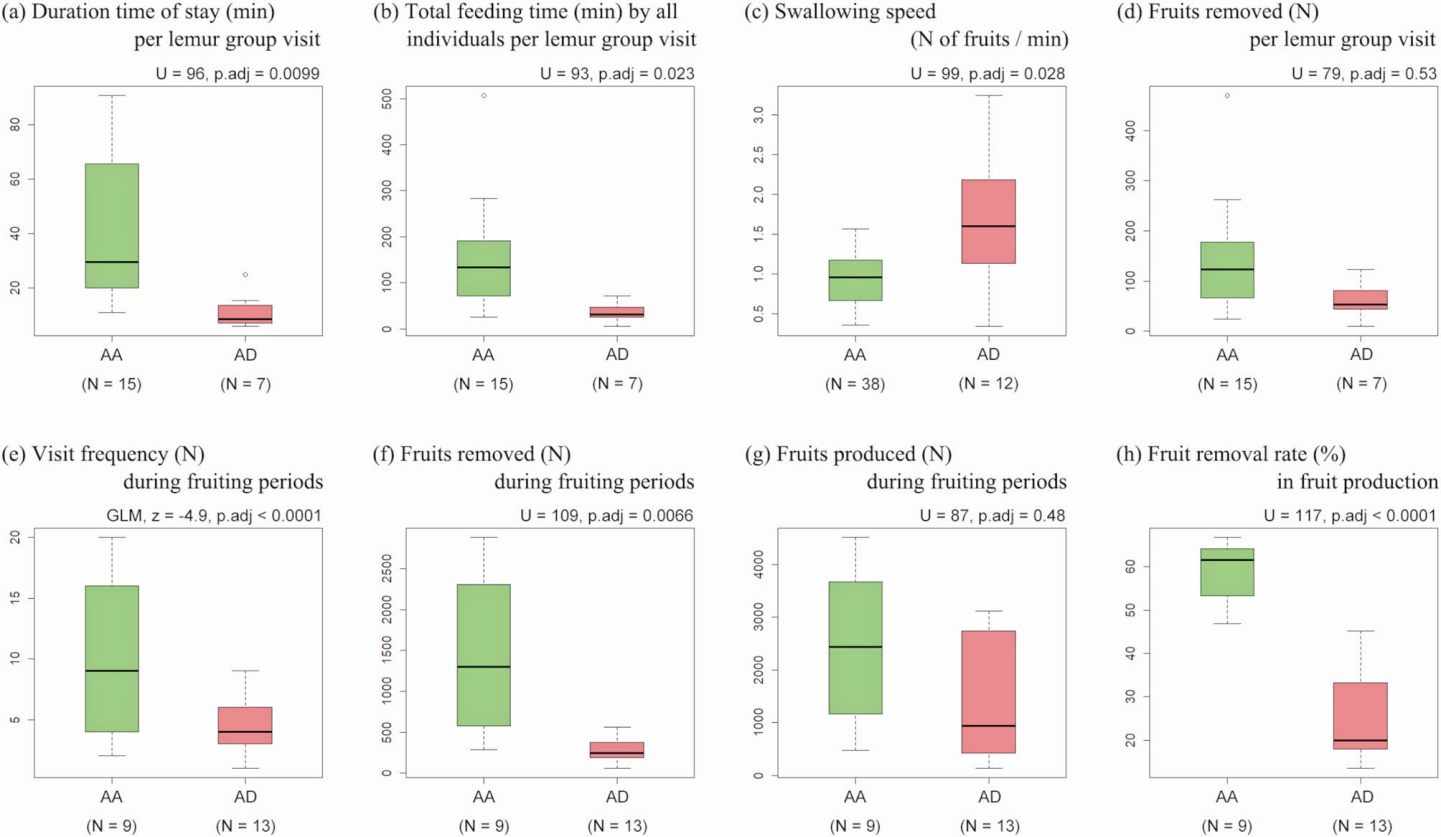
Comparison of fruit removal patterns by brown lemurs between *Astrotrichilia asterotricha* (*AA*) and *Abrahamia deflexa* (*AD*). (**a**) Duration time of stay in the tree crown per lemur group visit. (**b**) Total feeding time by all individuals per lemur group visit. (**c**) Swallowing speed by a lemur. (**d**) Fruit removed per lemur group visit. (**e**) Visit frequency during the fruiting period of focal trees. (**f**) Fruit removed during the fruiting period of focal trees. (**g**) Fruits produced during the fruiting period of focal trees. (**h**) Fruit removal rate in fruit production of focal trees. P values are Bonferroni-adjusted values. The sample sizes for (**a**), (**b**), and (**d**) are based on all visits (*AA*: N = 15 visits, *AD*: N = 7 visits), whereas those for (**c**) are based on observations of fruit swallowing speed (*AA*: N = 38 measurements, *AD*: N = 12 measurements) during focal tree observations. Sample sizes for (**e**)–(**h**) are based on the number of trees with at least one proxy for visit frequency (*AA*: N = 9 trees; *AD*: N = 13 trees) among the trees with fruit traps.

During the focal tree observations, I observed 15 and 7 instances of feeding activities on fruits by brown lemur groups at *AA* and *AD* trees, respectively. Then, fruit-swallowing speed was measured 38 times at *AA* trees and 12 times at *AD* trees. Lemur groups spent a longer time per visit in crowns of *AA* compared to *AD* (Fig. 2a). I observed 4 instances of lemurs staying at *AA* for longer than the shortest gut passage time of seeds (72 min^60, 86^; the longest stay was 90 min). No visits to *AD* were longer than 72 min. Once seed excretion under the crown of the focal tree during these prolonged visits is accounted for, I estimate that 99.99 ± SD 0.04% (N = 15) of the fruits consumed by lemur groups in *AA* were transported away from the crown (100% for *AD*). Although the total feeding time by all individuals per lemur group visit was longer in *AA* than *AD* (Fig. 2b), fruit swallowing speed was higher in *AD* than *AA* (Fig. 2c). The number of fruits removed per lemur group visit, which was estimated based on the mean fruit-swallowing speed and total feeding time by all individuals per lemur group visit (excluding the number of fruits defecated under the crown of the mother tree), did not differ significantly between the two tree species (Fig. 2d).

Brown lemur groups visited *AA* more frequently than *AD* (Fig. 2e). The frequent visits to *AA* trees led to the removal of larger numbers of *AA* fruits during the fruiting period (Fig. 2f). As the estimated number of fruits produced by focal trees did not differ between tree species (Fig. 2g), the fruit removal rate in the amount of fruit production was much higher for *AA* (mean ± SD, 58.8 ± 7.5%, N = 9) than *AD* (mean ± SD, 26.0 ± 11.5%, N = 13; Fig. 2h).

### Seedling occurrence in the post-dispersal phase

**Figure 3:**
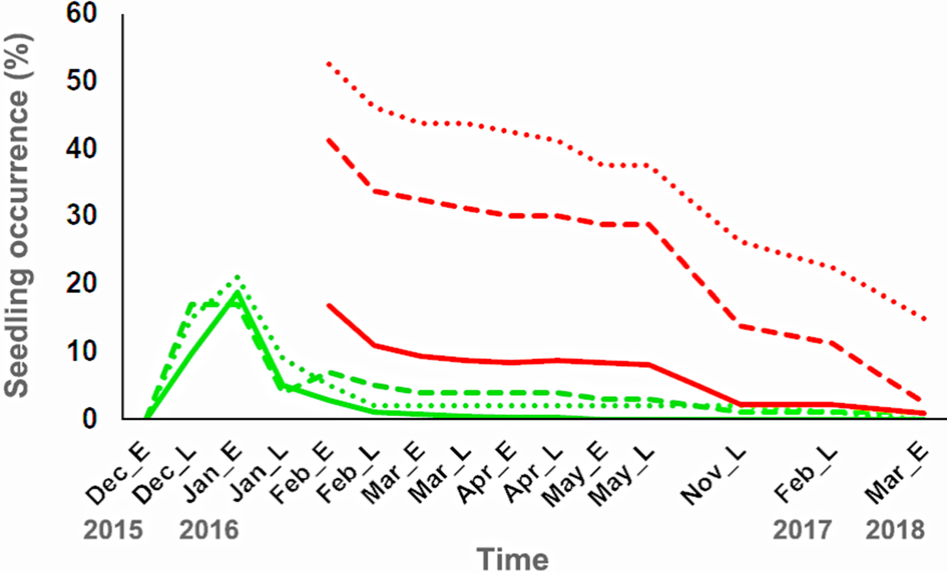
Temporal changes in seedling occurrence (%) of *Astrotrichilia asterotricha* (*AA*) and *Abrahamia deflexa* (*AD*). Solid green line = *AA* diaspores under the fallen condition (N = 400). Dashed green line = *AA* diaspores under the short-distance dispersal condition (N = 100). Dotted green line = *AA* diaspores under the long-distance dispersal condition (N = 100). Solid red line = *AD* diaspores under the fallen condition (N = 320). Dashed red line = *AD* diaspores under the short-distance dispersal condition (N = 80). Dotted red line = *AD* diaspores under the long-distance dispersal condition (N = 80). “E” and “L” on the x-axis refer to the early and late portions of each monitoring month, respectively.

Between the establishment of experimental quadrats in July–September 2015 and germination in late December 2015, 99.7% of the sown *AA* diaspores remained in the quadrats. Temporal changes in the occurrence of *AA* seedlings were similar among the three experimental conditions (Fig. 3). Although *AA* diaspores exhibited small peaks in seedling occurrence in late December 2015 and early January 2016, the seedlings, which were small and had epigeal cotyledons, were immediately consumed by predators such as rodents and herbivorous insects, or otherwise disappeared. Seedling occurrence rates were low from late January 2016.

To examine the contribution of seed dispersal by brown lemurs in post-dispersal phase, I used generalised linear mixed models (GLMMs) with a binomial distribution and logit-link function. The presence of live seedlings was the dependent variable and the density of diaspores per quadrat, distance from the mother tree, distance from the nearest conspecific fruiting tree, and canopy openness were independent variables. Moreover, the ID of the mother trees was treated as a random effect (see "Methods" for more information). In the GLMM for *AA*, the best models included no significant independent variables in early January 2016, when seedling occurrence peaked, or in late January 2016, when seedling numbers rapidly decreased (Table 3). Diaspore density negatively affected seedling occurrence as of the first rainy season until late April 2016. From the fruiting season (early May 2016), the distance from the nearest fruiting tree positively affected seedling occurrence. Based on the relative importance of each variable (IOV) of upper-ranked models and best models (Supplementary Table S3 online), canopy openness strongly and positively affected the successful seedling occurrence from late February 2016 to late February 2017.

**Table 3:** Best models of generalized linear mixed model analyses for the effects of distance to mother tree, distance to conspecific fruiting tree, density of diaspores and canopy openness on seedling occurrence of *Astrotrichilia asterotricha.*

Period	Intercept		Distance to mother tree		Distance to fruiting tree		Density of diaspores		Canopy openness		AIC
	Coefficient ± SE	*P* value	Coefficient ± SE	*P* value	Coefficient ± SE	*P* value	Coefficient ± SE	*P* value	Coefficient ± SE	*P* value	
Late December, 2015	− 1.67 ± 0.39	< 0.0001	Not selected		Not selected		− 0.040 ± 0.018	0.022	Not selected		417.3
Early January, 2016	− 2.75 ± 0.73	0.0002	Not selected		Not selected		Not selected		0.099 ± 0.057	0.084	569.1
Late January, 2016	− 3.09 ± 0.27	< 0.0001	0.0066 ± 0.0042	0.12	Not selected		Not selected		Not selected		257.9
Early February, 2016	− 2.66 ± 0.49	< 0.0001	Not selected		Not selected		− 0.056 ± 0.029	0.053	Not selected		194.6
Late February, 2016	− 8.98 ± 3.21	0.0052	− 0.016 ± 0.012	0.16	Not selected		− 0.16 ± 0.058	0.0065	0.51 ± 0.23	0.029	101.9
Early March, 2016	− 9.81 ± 3.30	0.0029	Not selected		Not selected		− 0.11 ± 0.051	0.029	0.49 ± 0.23	0.032	88.6
Late March, 2016	− 9.91 ± 3.44	0.0039	Not selected		Not selected		− 0.15 ± 0.060	0.013	0.53 ± 0.24	0.029	79.4
Early April, 2016	− 8.75 ± 3.62	0.016	Not selected		Not selected		− 0.18 ± 0.075	0.015	0.43 ± 0.25	0.086	70.1
Late April, 2016	− 8.75 ± 3.62	0.016	Not selected		Not selected		− 0.18 ± 0.075	0.015	0.43 ± 0.25	0.086	70.1
Early May, 2016	− 13.00 ± 3.55	0.0003	Not selected		0.13 ± 0.047	0.0049	Not selected		0.46 ± 0.22	0.036	51.7
Late May, 2016	− 13.00 ± 3.55	0.0003	Not selected		0.13 ± 0.047	0.0049	Not selected		0.46 ± 0.22	0.036	51.7
Late November, 2016	− 20.79 ± 6.98	0.0029	Not selected		0.21 ± 0.11	0.046	Not selected		0.82 ± 0.35	0.02	30.3
Late February, 2017	− 23.62 ± 9.09	0.0094	Not selected		0.15 ± 0.077	0.058	Not selected		1.09 ± 0.54	0.045	24.1

Temporal changes in *AD* seedling occurrence were highest among seeds dispersed over long distances, followed by those dispersed over short distances and fallen seeds (Fig. 3). Although the unprotected *AD* seeds were heavily predated by rodents and granivorous insects, the slight decreasing trends in seedling occurrence indicated that *AD* seedlings survived long after germination under all three experimental conditions. The GLMMs for *AD* indicated that diaspore density was significant, had the highest IOV, and negatively affected *AD* seedling occurrence until late November 2016 (Table 4; Supplementary Table S4 online). The best models selected distances from the fruiting trees and the mother trees as the sole independent variables with positive effects on survival of the 1-year-old (late February 2017) and 2-year-old seedlings (early March 2018), respectively. Although canopy openness was included in several of the best models (Table 4), it had a low IOV (Supplementary Table S4 online).

**Table 4:** Best models of generalized linear mixed model analyses for the effects of distance to mother tree, distance to conspecific fruiting tree, density of diaspores and canopy openness on seedling occurrence of *Abrahamia deflexa.*

Period	Intercept		Distance to mother tree		Distance to fruiting tree		Density of diaspores		Canopy openness		AIC
	Coefficient ± SE	*P* value	Coefficient ± SE	*P* value	Coefficient ± SE	*P* value	Coefficient ± SE	*P* value	Coefficient ± SE	*P* value	
Early February, 2016	− 1.31 ± 1.19	0.27	Not selected		Not selected		− 0.10 ± 0.016	< 0.0001	0.13 ± 0.086	0.14	501.7
Late February, 2016	− 2.35 ± 1.31	0.072	0.0061 ± 0.0043	0.16	Not selected		− 0.087 ± 0.024	0.0003	0.15 ± 0.094	0.11	434.1
Early March, 2016	− 1.92 ± 1.29	0.14	Not selected		Not selected		− 0.12 ± 0.018	< 0.0001	0.15 ± 0.094	0.11	411.2
Late March, 2016	− 0.49 ± 0.46	0.29	0.0071 ± 0.0042	0.095	Not selected		− 0.098 ± 0.025	< 0.0001	Not selected		400.8
Early April, 2016	− 0.56 ± 0.47	0.23	0.0072 ± 0.0043	0.093	Not selected		− 0.096 ± 0.025	0.0001	Not selected		394.6
Late April, 2016	− 0.56 ± 0.47	0.23	0.0065 ± 0.0043	0.13	Not selected		− 0.095 ± 0.025	0.0002	Not selected		397.7
Early May, 2016	− 0.16 ± 0.29	0.58	Not selected		Not selected		− 0.12 ± 0.018	< 0.0001	Not selected		390.0
Late May, 2016	− 0.15 ± 0.30	0.61	Not selected		Not selected		− 0.12 ± 0.018	< 0.0001	Not selected		384.4
Late November, 2016	− 1.61 ± 0.63	0.011	0.011 ± 0.0053	0.045	Not selected		− 0.12 ± 0.038	0.0016	Not selected		227.0
Late February, 2017	− 4.00 ± 0.53	< 0.0001	Not selected		0.073 ± 0.013	< 0.0001	Not selected		Not selected		208.9
Early March, 2018	− 4.69 ± 0.57	< 0.0001	0.029 ± 0.0059	< 0.0001	Not selected		Not selected		Not selected		125.7

### Cumulative probability from fruit production to 2-year-old seedling stages

**Figure 4:**
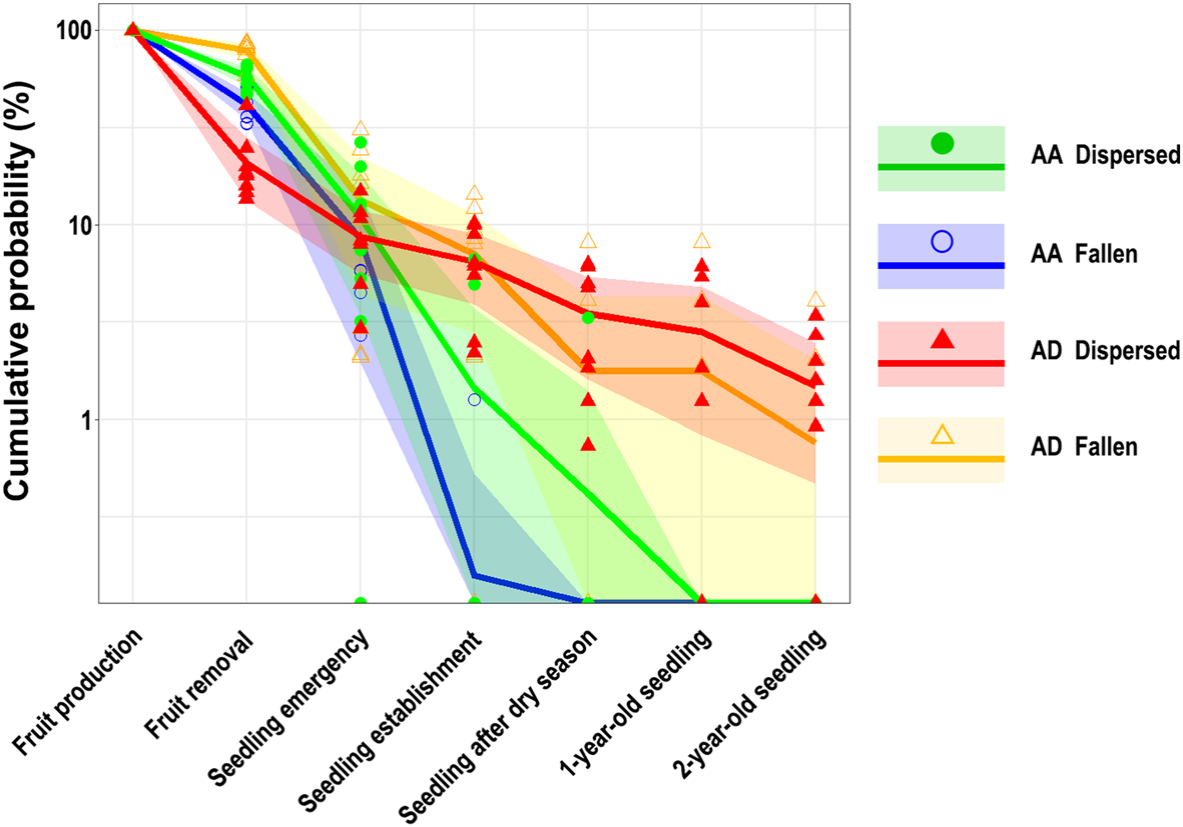
Cumulative probabilities (means ± 95% confidence intervals) of new recruitment from the fruit production to 2-year-old seedling stage in *Astrotrichilia asterotricha* (*AA*) and *Abrahamia deflexa* (*AD*) under conditions of seed dispersal by brown lemurs and falling under the crown of mother tree. Green line with filled circles = *AA* under the dispersed condition. Blue line with open circles = *AA* under the fallen condition. Red line with filled triangles = *AD* under the dispersed condition. Yellow line with open triangles = *AD* under the fallen condition. N = 8 trees for both species.

When comparing the temporal changes in cumulative probabilities of *AA* between the dispersed and fallen conditions (Fig. 4, green vs. blue lines), the probabilities at the stage of fruit removal were higher in dispersed condition than fallen condition (V = 33, *P* = 0.039). However, high mortality after the seedling emergence stage eliminated the differences in probabilities between the two conditions (*P* > 0.05). Seedlings in the fallen condition were dead after the dry season, whereas the seedlings under the dispersed conditions were dead by the 1-year-old seedling stage.

With respect to *AD* (Fig. 4, red vs. yellow lines), probabilities at the fruit removal stage were higher for fruits in the fallen condition (V = 0, *P* = 0.0078). However, because dispersed seeds avoided the high mortality caused by post-dispersal predation (see Fig. 3), the cumulative probabilities did not differ between the two conditions after the seedling emergence stage (*P* > 0.05). Although cumulative survival probability did not differ between the two conditions by the 2-year-old seedling stage, seedlings in the fallen condition died in six of the eight focal trees, whereas seedlings in the dispersed condition died in only two focal trees.

Temporal changes in cumulative probabilities under dispersal conditions varied between *AA* and *AD* (Fig. 4, green vs. red lines). Although the probabilities at the fruit removal stage were higher for *AA* than *AD* (U = 64, *P* = 0.00016), the probabilities at the seedling emergence stage did not differ between species (U = 35, *P* = 0.80). From the seed establishment stage onwards, the probabilities were lower for *AA* than *AD* (*P* < 0.01) and none of the *AA* seedlings survived more than 1 year.

## Discussion

High fruit removal rates by animals relative to the amount of fruit produced by the mother trees have been reported in previous studies. For example, spider monkeys removed 46.9% of *Virola calophylla* fruits^14^, similarly, 26 bird species removed 50.4–67.8% of *Prunus mahaleb* fruits^16^, 7 species of arboreal and terrestrial mammals removed 60% of *Platymitra macrocarpa* fruits^12^, 3 primate species removed 85% of *Monodora myristica* fruits^13^, and 2 primate species removed 86% of *Salacia chinensis* fruits^8^. This study demonstrated that the brown lemur is the sole effective seed disperser for both *AA* and *AD*, whereas a small number of other fruit consumers (*Cheirogaleus medius*, *Lepilemur edwardsi*) do not contribute to seed dispersal due to the large size of the diaspores. However, the fruit removal rates differed significantly between the two tree species, i.e. 58.8% for *AA* compared to 26.0% for *AD*, despite their reliance on the same seed dispersal agent.

The high removal rate for *AA* was biased towards more frequently visited trees with larger crowns, a higher density of fallen fruits, and a greater number of adjacent fruiting trees. This pattern of tree selection is similar to the patterns observed for woolly monkeys (*Lagothrix lugens*), which select large trees with clumped distributions^18^. The selection of large fruit patches corresponds to a foraging theory to minimise costs to travel between fruit patches^17, 18^. Moreover, large fruit patches can mitigate within-group feeding competition^18, 20, 57^. During the dry season, which corresponds to the fruiting period of *AA* in Ankarafantsika, the diversity of fruit resources is lower than during the rainy season^56^. Moreover, low water availability constrains the activity of brown lemurs^58, 59^ to short daily path lengths (469 m on average) to minimise energy and water costs^60^. Under such conditions, repeated and prolonged visits to clusters of *AA* trees with large fruit crops likely represent the best foraging strategy for this species.

By contrast, during the rainy season, when fruit resources are diversified and water is abundant^56^, brown lemurs generally become more active^58, 59^ and travel longer distances to visit various fruiting trees (average daily path length = 1172 m)^60^; this can be interpreted as an active foraging strategy that maximises fruit acquisition^61, 62^. From the plant perspective, because competition among trees for acquiring visitation opportunities by seed dispersers increases with the increased diversity and abundance of fruiting trees during the rainy season^56^, *AD* did not experience the repeated and prolonged visits by brown lemurs that *AA* did. Consequentially, seasonal changes in the foraging strategies of brown lemurs create a gap in fruit removal rates between the dry-season-fruiting *AA* and rainy-season-fruiting *AD*.

The two tree species exhibited different patterns of seedling recruitment. As argued in previous studies^63, 64^, these distinctive patterns would be linked to the functional traits of seeds and seedlings. Considering the function of seed protection^65^, the robust endocarp of *AA* fruit probably facilitated avoidance of devastating post-dispersal seed predation during the long period before the rainy season, and *AA* exhibited similar peaks in first seedling occurrence under all experimental conditions (as shown by the GLMM for early January 2016). However, these peaks were uniformly low, likely due to pre-dispersal seed predation. Indeed, I observed adult weevils (Curculionoidea species) appearing from ungerminated diaspores between February and April 2016. Weevils are well known pre-dispersal predators of seeds with physical protection^66, 67^. Due to the small investment of *AA* in seed nutrients, the strategy of small *AA* seedlings is to promote growth via photosynthesis by the epigeal cotyledons. Following germination, the exposed, nutrient-rich cotyledons were immediately targeted by predators (Fig. 3). Cotyledon removal typically leads to mortality in seedlings of phanerocotylar epigeal species (in which the cotyledons emerge from the seed coat aboveground) with foliaceous cotyledons^68^. After the rapid decline in seedlings, the high density of diaspores during the first rainy season and proximity to the nearest fruiting trees during the following fruiting season have a gradual negative impact on the surviving seedlings owing to Janzen-Connell effects. However, the best GLMM did not select any Janzen-Connell variables as significant at the end of the monitoring period in the second rainy season. By contrast, canopy openness exhibited positive effects on seedling occurrence, corresponding to the strategy of seedlings with epigeal cotyledons which require high light levels for photosynthesis^63^.

In contrast to *AA*, *AD* has a large seed including cryptocotylar hypogeal cotyledons (in which the cotyledons remain underground within the seed coat), with reserve storage but no physical protection. Some large-seeded species exhibit weaker distance-dependent effects on seed survival due to the low mortality rates of large seeds^69^. However, because sites with aggregations of unprotected seeds are attractive feeding patches to seed predators, the probabilities of *AD* seedling occurrence were considerably restricted by seed density from the first monitoring period in early February 2016. Among the main predators of *AD* seeds were Dermestidae beetle species, for which I found larvae and adults (identified by the Botanical and Zoological Garden of Tsimbazaza). Considering density-dependent predation, these beetles likely invaded the seeds during the post-dispersal stage^67^. Following germination, *AD* grows quickly into large seedlings by exploiting the rich nutrient investment in the reserve storage cotyledons and predators often attacked the cryptocotylar hypogeal cotyledons but not main body of the seedling even after germination. In general, large seedlings have high survival rates^70^, and removal of the reserve-storage cotyledons does not severely impact survival once true leaves have developed^71^. These general patterns were consistent with those observed in *AD*, and the minor decrease in seedling occurrence maintained the Janzen-Connell effects until the final monitoring of 2-year-old seedlings. Interestingly, in the GLMMs for both tree species, the distance from fruiting trees became more important than the density of diaspores in the experimental quadrats from the fruiting season in the year following germination (early May 2016 for *AA*, late February 2017 for *AD*). This suggests the presence of Janzen-Connell effects on the 1-year-old seedlings associated with the nearest fruiting trees^72, 73^.

This study highlighted the differential role of brown lemurs in the seed dispersal and recruitment strategies of *AA* and *AD*. For *AA*, although numerous of seeds were dispersed by brown lemurs, high seedling mortality undermined overall recruitment success. This modification of the SDE during the post-dispersal phase is consistent with the findings of several other studies^13, 31–33^. However, considering the high quantity of fruit removal and improved survival rates in high-light microhabitats, *AA* likely leads to successful colonisation of suitable sites via seed dispersal by brown lemurs. A study simulating plant population dynamics over 1000 years suggested that quantitative seed dispersal by an animal species into safe sites can maintain plant populations despite the high mortality (99.86%) of deposited seeds or seedlings^74^. The contribution of seed dispersal by brown lemurs to reproductive success, and population regeneration in *AA* could similarly be confirmed by simulating seed dispersal, seedling establishment, and sapling growth and survival over numerous generations, while also accounting for gap formation.

Conversely, as Janzen-Connell effects are often seen for large-seeded plants^75^, *AD* seeds that escaped from the dangerous zones, i.e. areas with high densities of fallen seeds near the mother and fruiting trees, exhibited effective seedling establishment. This successful recruitment improved the SDE of brown lemurs for *AD* even though the fruit removal rate was relatively low. Although the cumulative probability of *AD* seedlings at the 2-year-old seedling stage was similar between seeds in the fallen and dispersed conditions (Fig. 4), higher recruitment rates can be expected for dispersed seeds when subsequent stages are considered. That is, brown lemurs alleviate seed dispersal limitation for *AA* via their contribution to the quantitative component of SDE, whereas they alleviate the seedling recruitment limitation of *AD* via their contribution to the qualitative component. To understand how the SDE of brown lemurs contributes to the population structures and regeneration patterns of *AA* and *AD*, it is necessary to examine the consequences of the cumulative effectiveness in older generations, from saplings to young trees^13, 76^. Moreover, various combinations of animal behavioural strategies and seed/seedling functional traits are likely responsible for the species-specific patterns seen in tree recruitment, even though many plant species share the same dispersal agents in forest ecosystems. Scaling up SDE studies to the community level by considering the interactions between animal behaviour and plant traits will improve our understanding of the contribution of seed dispersal to plant biodiversity.

Large frugivores are thought to accelerate reforestation via their role as effective restoration agents, because they disperse large quantities of seeds for a wide variety of plant species, including those with large seeds, over broad areas^42^. In recent years, selective afforestation of large-seeded plant species has been proposed as a strategy to attract and introduce large frugivores to reforestation sites^77^, and in Madagascar afforestation using plants on which lemurs forage is currently being discussed^78^. My findings provide important insight that could facilitate the design of reforestation plans. For example, lemurs may be attracted by large fruiting patches, which can be created by agglomerating a range of tree species that fruit in the same season, thus improving SDE during the seed dispersal phase. To strengthen SDE during the post-dispersal phase, tree species with large, unprotected seeds should be planted in a scattered manner to weaken the Janzen-Connell effects, whereas tree species with epigeal cotyledons must be planted in high-light habitats. To exploit the interactions between large frugivores and large-seeded plants, which are vulnerable to anthropogenic disturbance, as a reforestation tool, it will be necessary to obtain a deeper understanding of seed dispersal by animals in plant regeneration strategies^79^.

## Materials and methods

### Study site

The study site was Ankarafantsika National Park in northwestern Madagascar. I conducted surveys in Jardin Botanique A (JBA), a rectangular grid trail system designated as a research area, at Ampijoroa Forest Station in a primary dry deciduous forest. The mean ± SD hourly temperature and humidity for the 3-year study period from May 2015 to April 2018 were 25.7 ± 4.3 °C and 68.1 ± 22.6%, respectively, as measured using a datalogger (RTR-53A; T&R, Nagano, Japan) at the centre of JBA (16°19′S, 46°48′E). It rarely rained during the dry season (May–October: 5.2 ± SD 4.5 mm) and most rainfall occurred during the rainy season (November–April: 1371.9 ± SD 158.3 mm) over the 3-year study period (Supplementary Fig. S1 online).

### Study subjects

Based on known species of seed dispersers and their distributions ^43, 48, 51, 80–82^, 10 vertebrates were recognised in the JBA disperser assemblages, i.e. 3 species of birds (*Treron australis*, *Alectroenas madagascariensis*, *Hypsipetes madagascariensis*), 3 species of fruit bat (*Pteropus rufus*, *Eidolon dupreanum*, *Rousettus madagascariensis*), 3 species of small-bodied nocturnal lemur (*Cheirogaleus medius*, *Microcebus murinus*, *Microcebus ravelobensis*) and 1 species of large-bodied lemur (the common brown lemur: *Eulemur fulvus*). Although another congeneric lemur (mongoose lemur: *Eulemur mongoz*) occurred in Ankarafantsika National Park, none inhabited the JBA. In this forest, the common brown lemur (Fig. 1a) has been suggested to be the sole seed disperser for large seeds > 10 mm in diameter^48^. This primate weighs 1773.2 ± SD 223.4 g (N = 11 captured in JBA; Sato unpublished) and shows cathemeral activity patterns^59^. During the periods of focal tree observation (see below), two groups consisting of 20 individuals without infants ranged in JBA^59^.

This study focused on two large-diaspore tree species. I selected *Astrotrichilia asterotricha* (*AA*), a Madagascan endemic tree species that bears fruit during the dry season. This fruit covered with green fresh pulp (fruit size: 17.5 × 19.8 × 19.0 mm, 3.8 g, N = 255) contains a large diaspore covered with a hard shell (diaspore size: 15.4 × 17.8 × 16.9 mm, 2.8 g, N = 255) that includes 1–3 seeds (average 1.2 ± SD 0.5 seeds, N = 120 diaspores) that are small in size (10.4 × 6.8 × 1.6 mm, 0.065 g, N = 20; Fig. 1b). This species represents the main fruit resource for brown lemurs and the large diaspores are frequently dispersed by these animals during the dry season^56, 83^. This plant establishes small seedlings with phanerocotylar epigeal foliaceous cotyledons (Fig. 1c). I also selected a rainy-season-fruiting tree species, *Abrahamia deflexa* (*AD*), which is also endemic to Madagascar. The pink fruit (23.8 × 15.3 × 15.0 mm, 3.3 g, N = 202) contains a single large seed without physical protection (23.0 × 14.0 × 13.7 mm, 2.7 g, N = 202; Fig. 1d). The fruits are swallowed and dispersed by brown lemurs during the rainy season^56, 83^. This species establishes a large cryptocotylar hypogeal seedling with reserve-storage cotyledons (Fig. 1e).

### Focal tree observation

To estimate fruit removal by seed disperser assemblages, I observed their visits to the fruiting crowns and their fruit-eating behaviour at the focal fruiting trees. In a plot of 13.5 ha (450 m in east–west direction and 300 m in north–south direction) established at the centre of JBA, I tagged all adult trees of both species with diameters at breast height (DBH) > 10 cm with number plates and located with a Global Positioning System (GPS) device (GPSMAP 62 s; Garmin International, Olathe, KS, USA) until March 2015 (328 individuals of *AA* and 241 individuals of *AD* in the 13.5 ha; Supplementary Fig. S2 online). Tree densities suggest that the populations of these species have successfully regenerated. Before maturation of the fruits of each species, I examined the fruit production and visibility of each crown to determine which individual trees should be observed. Then, I selected 7 individuals of each species as focal trees for observation based on high visibility for observation and various level of fruit production to examine its effect on fruit removal^14^. I included 2 additional individuals of *AA* outside of, but near to, the 13.5 ha plot due to its high visibility. In total, 9 individuals of *AA* were observed. To ensure independent sampling, only trees whose crown did not overlap with those of other conspecific fruiting trees were selected for observation.

During the fruiting periods of each focal tree, I observed frugivores from a blind tent placed under the crown of the focal trees. To detect frugivorous activities of diurnal and nocturnal animals, observations were performed over four time sessions: morning (03:00–08:00), day (09:00–14:00), evening (15:00–20:00) and night (21:00–02:00). I conducted nocturnal observations using a headlamp covered with red cellophane to avoid disturbing the nocturnal animals^83^. Two cycles of these four sessions (40 h observation) were conducted for each focal tree and I observed *AA* and *AD* for a total of 360 and 280 h, respectively. I identified all animals entering into or beneath the crown and recorded the duration of each visit in seconds. When the visitors clustered in groups, such as for gregarious lemurs, the number of visitors within the focal crown was monitored in seconds during the group visitation. The duration of group visit was measured as the time from arrival of the first individual to departure of the last individual. For the animals under good conditions for observation (Supplementary Movie S1 online), I evaluated seed handling behaviour, the number of fruits swallowed and the duration of focal observations to calculate swallowing speed^14^.

### Fruit traps

Fruit traps were made in inverted cones of mosquito netting (depth = 80 cm) with a circular mouth of polyethylene pipe (receiving face = 0.5 m^2^). Each trap was supported by 3 vertical woody poles at a height of 1 m above the ground. I installed traps below the crowns of 13 trees of each species, including the above focal trees for observation. To ensure independent sampling, I only selected trees with crowns that did not overlap with those of other conspecific fruiting trees. I measured crown radius along 8 rays and used the mean of the 8 measurements to calculate a circular area approximating the horizontal crown area of each target tree^84, 85^. Several traps were placed for each tree that covered over 5% of the crown area (3–11 traps for *AA*, 3–7 traps for *AD*) and I counted the fruits that fell into the traps twice a month during the fruiting period of each tree. I calculated the densities of fallen fruits as the total number of counted fruits fallen into the traps divided by the total area of fruit traps. Moreover, I estimated the number of fallen fruits in each focal tree based on the product of the density of fallen fruits and the crown area.

As brown lemurs, the expected main seed dispersers, frequently defecated during their activities^48, 60^, the fruit traps caught faeces defecated by members of lemur groups and the presence of faeces was taken as evidence of lemur visits. I recorded the presence of brown lemur faeces in the fruit traps every 2 days during the fruiting periods. I confirmed the faeces of brown lemurs by size, smell, material traits and contents based on personal experience^48^. This information was used as a proxy of visit frequency by brown lemurs to the focal trees.

### Measurement of tree traits

To understand brown lemurs’ selection of fruiting trees for visitation, I measured 4 traits in the 13 trees with fruit traps in each species: crown area, density of fallen fruits, fresh weight of pulp per fruit and the number of adjacent fruiting trees. The measurement of crown area (m^2^) and density of fallen fruits (number/m^2^) are described above. I measured pulp weight (g) as the difference between fruit mass and seed mass for each fruit in fresh and good condition using an electronic scale (precision: 0.05 g). I calculated the mean of pulp weight from 9 to 23 fruits for each focal tree^14, 17^. During the fruiting period of each focal tree, I counted the number of fruiting trees > 10 cm in DBH within a 30 m radius^15^. I identified all tree species that provided fruit resources to brown lemurs based on the list of the fruits in their diet in Ankarafantsika^48, 56^.

### Fruit removal rate

Fruit removal rate represents the percentage of fruits removed from the crown by animals relative to the amount of fruit produced by the mother tree^14^. I calculated the removal rate based on the number of removed fruits divided by the sum of removed and fallen fruits. This study focused on fruit removal by brown lemurs because there were no other effective seed dispersers for either of the focal tree species (see also Results). I calculated the number of removed fruits based on the product of swallowing speed, total feeding time by all individuals per lemur group visit (sum of feeding time by all lemur individuals in a group visit), and visit frequency. Brown lemurs begin eliminating swallowed seeds 72 min after the feeding^60, 86^. If the duration time of their stay in the tree crown per visit by a lemur group is longer than 72 min, seeds swallowed prior to the timing 72 min before the end of the stay may be excreted under the crown. The longest observed visit was 90 min (see Results). As the probability that a seed swallowed by a brown lemur will be eliminated after 72–90 min is 0.21%^60, 86^, I assumed that 0.21% of the number of fruits swallowed prior to 72 min before the end of the stay would be excreted under the crown of the focal tree, and excluded them from calculations of the number of removed fruits. The number of fallen fruits was obtained as the product of the density of fallen fruits and the crown area. As fruit-eating activities of brown lemurs could not be observed in all focal trees, I used the mean values of swallowing speed and total feeding time by all individuals per lemur group visit for each tree species.

### Monitoring experiment of seedling establishment

To examine seedling establishment of the two tree species, I set up an experiment under the conditions of seeds fallen under mother trees and seeds dispersed by animals for 10 *AA* trees (both fruit trapping and focal tree observation for 8 trees and only fruit trapping survey for 2 trees) and 8 *AD* trees (both fruit trapping and focal tree observation for 7 trees and only fruit trapping survey for 1 tree). To replicate the conditions of fallen seeds, I placed 20 fruits in a quadrat (50 × 50 cm) under the crown of the focal tree. To mimic the conditions of seeds dispersed over a short distance by brown lemurs, I placed 5 diaspores with pulp removed by hand and covered with 5 g brown lemur faecal material, considering their small faeces^48^, in a quadrat (50 × 50 cm) 20 m from the trunk of the focal tree measured using a GPS device. To replicate the conditions of seeds dispersed at a long distance by brown lemurs, 5 diaspores with pulp removed and covered with 5 g brown lemur faecal material were placed in a quadrat (50 × 50 cm) 100 m from the trunk of the focal tree measured using a GPS device. Two quadrats were set up for each condition for each focal tree. In total, I collected 600 *AA* diaspores and 480 *AD* seeds from the intact and ripe fruits that had fallen under the crowns of focal trees during the fruiting seasons of each species and placed them in the experimental quadrats with numbered flags for identification. Following quadrat establishment (July–September 2015 for *AA*, January–February 2016 for *AD*), I monitored the quadrats twice a month (around the 10th and 25th of each month) and recorded the occurrence of seedlings or roots until late May 2016 to monitor seedling establishment in the first rainy season. They were monitored again in late November 2016 after the first dry season for the seedlings, late part of February 2017 in the second rainy season and the early part of March 2018 in the third rainy season.

To take the Janzen-Connell effects into consideration, I measured the distances from the seedling quadrats to the nearest conspecific fruiting trees^28^ using GPS and QGIS 2.18 with the database of all fruiting trees of those two species (Supplementary Fig. 2S online). To examine the effects of light conditions in each quadrat, I took hemispheric photographs with a fish-eye lens (Opto Canopy 180; Taisei Fine Chemical Co., Ltd., Chiba, Japan) at the height of 1 m above ground level and evaluated canopy openness (%) as the proportion of sky area in photographs using CanopOn version 2.03 software (http://takenaka-akio.org/etc/canopon2/).

### Data analyses

#### Fruiting tree selection

I examined the selection of fruiting trees for visitation by brown lemurs using GLMs with a Poisson distribution and log-link function treating the proxy of visit frequency as a dependent variable and crown area, density of fallen fruits, fresh weight of pulp per fruit and the number of adjacent fruiting trees as independent variables. In this analysis, the length of the fruiting period was treated as an offset term to eliminate bias of frequencies for checking the fruit traps. GLMs were fitted using R version 3.6.3 (R Foundation for Statistical Computing, Vienna, Austria).

#### Comparison of fruit removal patterns between tree species

To understand species-specific patterns of fruit removal by brown lemurs, I compared the following 8 variables between the two tree species using the Mann–Whitney U test: duration time of stay in the tree crown per lemur group visit, total feeding time by all individuals per lemur group visit, swallowing speed, number of fruits removed per lemur group visit, proxy of visit frequency during the fruiting period, number of fruits removed during the fruiting period, number of fruits produced during the fruiting period, and fruit removal rate in the amount of fruit production. The first 4 variables were analysed based on the data evaluated for all visits (*AA*: N = 15 visits, *AD*: N = 7 visits) or observations of swallowing speed (*AA*: N = 38 measurements, *AD*: N = 12 measurements) during the focal tree observations. The latter 4 variables were analysed based on the data estimated in each tree with one or more proxies of visit frequency (*AA*: N = 9 trees, *AD*: N = 13 trees) among the individual trees with fruit traps. Due to multiple comparisons, significant differences were identified based on Bonferroni-adjusted *P* values.

#### Janzen-Connell effects and effects of light conditions on seedling establishment

I examined the contribution of seed dispersal by brown lemurs on post-dispersal seed fate using GLMMs with a binomial distribution and logit-link function treating the presence of living seedlings (1 or 0) as a dependent variable and density of diaspores in a quadrat (20 or 5), distance from the mother tree (0, 20 or 100 m), distance from the nearest conspecific fruiting tree and canopy openness as independent variables. In GLMM analyses, the ID of the mother trees was treated as a random effect. To avoid the multicollinearity problem, GLMM analyses did not use the pairs of independent variables with high variance inflation factor (VIF) above the cut-off value (= 4.0)^87^, such as the pairs of distance from the mother tree and distance from the nearest fruiting tree in *AA* (VIF = 4.0) and *AD* (VIF = 7.2) and the pairs of density of diaspores and distance from the nearest fruiting tree in *AA* (VIF = 5.9) and *AD* (VIF = 6.1). I assessed the Akaike Information Criterion (AIC) in models with all combinations of independent variables to identify the best approximating model with the smallest AIC^88^ and good approximating models with ΔAIC (difference between AICs of the best and the targeted models) < 2.0^89^. To estimate the relative importance of each variable (IOV), Akaike weights of best and good models were summed for each independent variable^88, 90^. I fitted the GLMMs using the glmmML package and Akaike weights were calculated using the MuMIn package in R version 3.6.3.

#### Cumulative probability from fruit production to 2-year-old seedling stages

To evaluate the SDE by brown lemurs on seedling establishment in the two tree species, I calculated cumulative probabilities at the 6 stages, from fruit production to 2-year-old seedlings^23, 35^. For each tree species, cumulative probabilities were calculated separately for dispersed (including short- and long-distance dispersal) and fallen conditions.

- *Stage 0* The amount of fruit production was assumed to be 100%.
- *Stage 1* The probability of fruit removal by brown lemurs or the probability of falling under the mother trees in the amount of fruit production.
- *Stage 2* The probability of seedling emergence from dispersed or fallen diaspores in the amount of fruit production. This was calculated as the product of the Stage 1 probability and the probability of seedling occurrence under dispersal or fallen conditions at the emerging peaks (early January 2016 for *AA*, early February 2016 for *AD*).
- *Stage 3* The probability of seedling establishment of dispersed or fallen diaspores in the amount of fruit production. This was calculated as the product of the Stage 1 probability and the probability of seedling occurrence under the dispersal or fallen conditions at the end of the first rainy season (late April 2016).
- *Stage 4* The probability of seedling survival for dispersed or fallen diaspores in the amount of fruit production after the dry season. This was calculated as the product of the Stage 1 probability and the probability of seedling occurrence under dispersal or fallen conditions in late November 2016.
- *Stage 5* The probability of 1-year-old seedling survival of dispersed or fallen diaspores in the amount of fruit production. This was calculated as the product of the Stage 1 probability and the probability of seedling occurrence under dispersal or fallen conditions in late February 2017.
- *Stage 6* The probability of 2-year-old seedling survival of dispersed or fallen diaspores in the amount of fruit production. This was calculated as the product of the Stage 1 probability and the probability of seedling occurrence under dispersal or fallen conditions in early March 2018.

I calculated these probabilities in the individual trees that were also used in evaluation of fruit removal rates and experimental monitoring of seedling establishment. This analysis excluded trees with no proxy of visit frequency; therefore, I analysed 8 trees of *AA* and 8 trees of *AD*. For each tree species, I calculated cumulative probabilities at each stage and compared the dispersed and fallen conditions using Wilcoxon signed-rank tests. Moreover, I compared the probabilities at each stage between the two tree species with the Mann–Whitney U tests.

### Ethics approval

The field research complied with the protocols and principles for the Ethical Treatment of Non-Human Primates was approved by the Primate Research Institute of Kyoto University (KUPRI), Japan, and adhered to the legal requirements of the association *Madagascar National Parks* (MNP) and the research permissions authorized by the *Ministère de l’Environnement de l’Ecologie de la Mer et des Forêts Madagascar* (N°124 and 380 in 2015, N°250 in 2016 and N°287 in 2017). All methods of this study were performed in accordance with the institutional, national and international guidelines and regulations. Although *Eulemur fulvus* and *Abrahamia deflexa* are classified as Vulnerable on the IUCN Red List, all methods were conducted in accordance with the IUCN Policy Statement on Research Involving Species at Risk of Extinction so as not to disadvantage those species, but rather to contribute to their conservation.

## Supplementary Information

Supplementary Information 1.

Supplementary Movie S1.

## Data Availability

The datasets generated during and/or analysed during the current study are available from the corresponding author on reasonable request.
