# Supplementary material for: Significance of seed dispersal by the largest frugivore for large-diaspore trees
Source: Sci Rep. 2022 Nov 21;12:19086. doi: 10.1038/s41598-022-23018-x (PMC9678871; doi:10.1038/s41598-022-23018-x)
Supplement: Supplementary file 1 — Supplementary Information 1. [file 41598_2022_23018_MOESM1_ESM.pdf]

Supplementary Information

Journal: Scientific Reports
Title: Significance of seed dispersal by the largest frugivore for large-diaspore trees
Author: Hiroki Sato
Year: 2022

Legend of Movie S1.
Fruit-foraging behaviour in a tree of *Astrotrichilia asterotricha* during a night session of the focal tree observation. Movie S1 is available on the website of this research article as a supplemental video.

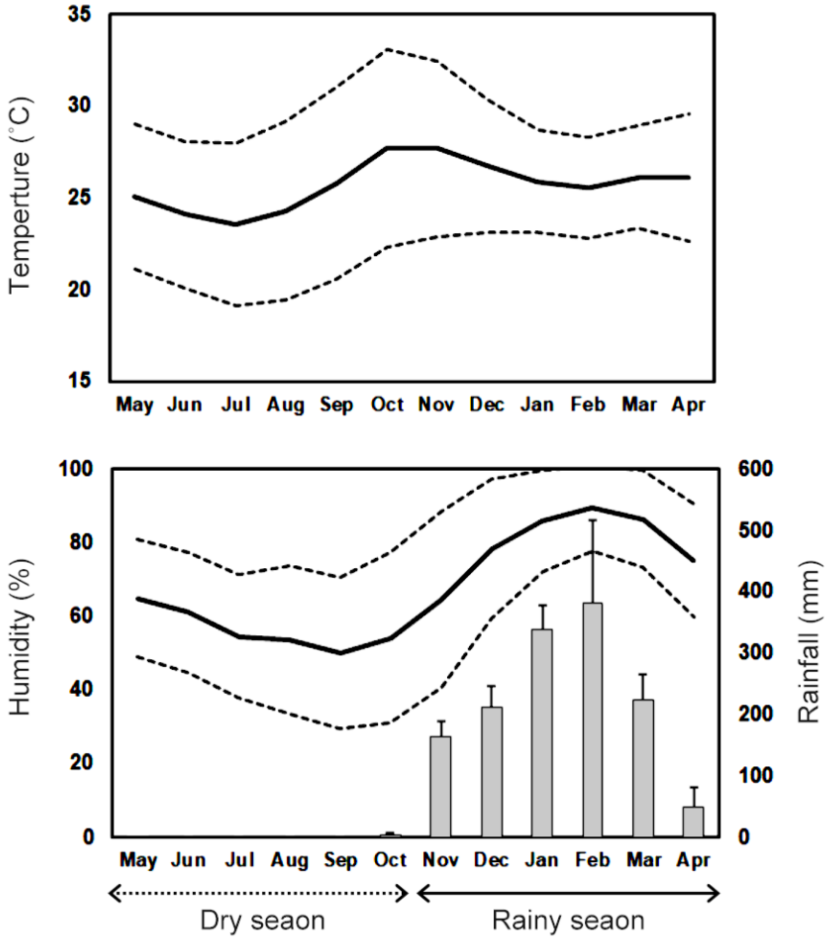

Fig. S1 Climate in Ankarafantsika National Park during the study period (May 2015–Apr 2018). Lines in the upper graph = mean hourly temperature (°C) mean SD for hourly temperature in each month. Lines in the lower graph = mean hourly humidity (%) and mean SD for hourly humidity in each month. Grey bars in the lower graph: mean and SD of monthly rainfall (mm).

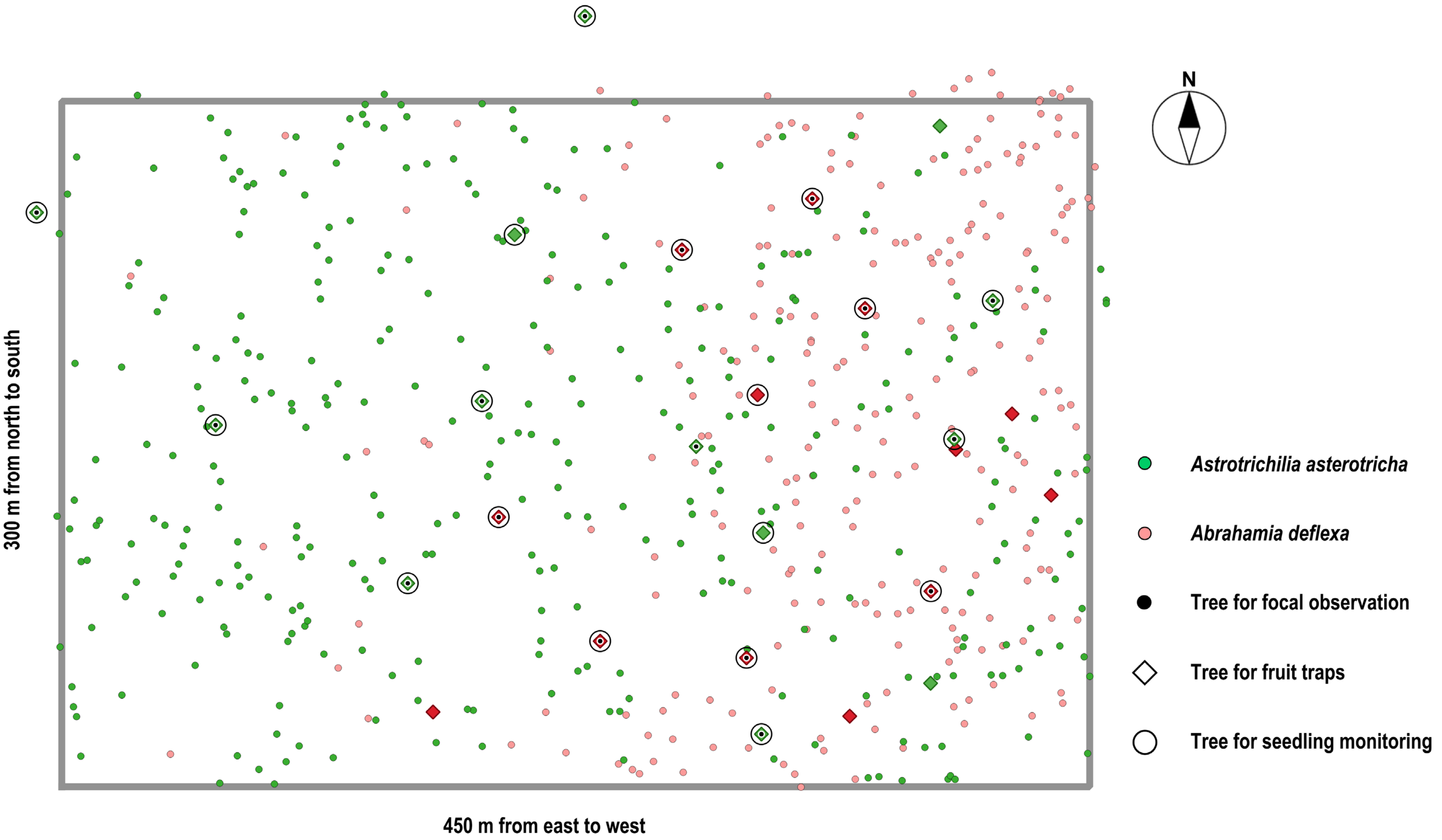

Fig. S2 Distribution of mature trees (≥ 10 cm in DBH) and targeted for research in *Asterotrichilia asterotricha* and *Abrahamia deflexa* in 13.5 ha plot (450 m × 300 m) in Ankarafantsika National Park. 328 individuals of *AA* and 241 individuals of *AD* grew inside the plot. In addition, 9 individuals of *AA* and 10 individuals of *AD* are marked outside the plot in this map

Table S1. Percentage of total time spent by individuals of each visiting species at *Astrotrichilia asterotricha* (*AA*). Total time spent by all individuals was 55.8 h. Time session indicates the time period when each species visited: M = morning, D = day, E = evening, N = night.

Species	Behavior	Time session	Total time spent by individuals (%)
Mammals			
Brown lemur (*Eulemur fulvus*)	Feeding on *AA* fruits	M, E, N	69.6
	Resting, Traveling, Vocalizing, Social behavior		24.9
Milne-edwards's spotive lemur (*Lepilemur edwardsi*)	Feeding on *AA* fruits	M, E, N	1.7
	Traveling, Feeding on leaves of other tree species		0.4
Coquerel's sifaka (*Propithecus coquereli*)	Traveling	M	0.1
Western tuft-tailed rat (*Eliurus myoxinus*) ?	Traveling	M	0.0
Birds			
Madagascar magpie-robin (*Copsychus albospecularis*)	Traveling, Feeding on insects, Searching insects	M, D, E	1.5
Coquerel's coua (*Coua coquereli*)	Traveling, Searching insects	D	0.4
Red-capped coua (*Coua ruficeps*)	Traveling, Searching insects	D, E	0.3
Long-billed greenbul (*Bernieria madagascariensis*)	Traveling, Searching insects	M, E	0.3
Crested drongo (*Dicrurus forficatus*)	Resting, Searching insects	M, D, E	0.2
Crested coua (*Coua cristata*)	Resting, Traveling, Feeding on insects	M, D, E	0.2
Rufous vanga (*Schetba rufa*)	Traveling, Searching insects	M, E	0.2
Madagascar paradise flycatcher (*Terpsiphone mutata*)	Traveling	D, E	0.1
Sickle-billed vanga (*Falculea palliata*)	Traveling	M, D	0.0
Greater vasa parrot (*Coracopsis vasa*)	Traveling	M	0.0
Total			100.0

Table S2. Percentage of total time spent by individuals of each visiting species at *Abrahamia deflexa* (*AD*). Total time spent by all individuals was 7.7 h. Time session indicates the time period when each species visited: M = morning, D = day, E = evening, N = night.

Species	Behavior	Time session	Total time spent by individuals (%)
Mammals			
Brown lemur (*Eulemur fulvus*)	Feeding on *AD* fruits	M, E	39.6
Fat-tailed dwarf lemur (*Cheirogaleus medius*)	Feeding on *AD* fruits	E, N	30.9
	Resting, Traveling		1.6
Milne-edwards's spotive lemur (*Lepilemur edwardsi*)	Resting, Traveling, Feeding on leaves of other tree species	M, N	15.4
Coquerel's sifaka (*Propithecus coquereli*)	Resting	M	2.7
Mouse lemur (*Microcebus murinus* or *M. ravelobensis*)	Feeding on *AD* fruits	M	0.6
Common tenrec (*Tenrec ecaudatus*)	Traveling	D	0.1
Birds			
Red-capped coua (*Coua ruficeps*)	Traveling, Searching insects	M, D, E	4.2
Broad-billed roller (*Eurystomus glaucurus*)	Resting, Vocalizing	M, D	1.7
Rufous vanga (*Schetba rufa*)	Traveling	M	1.4
Madagascar paradise flycatcher (*Terpsiphone mutata*)	Resting, Vocalizing	M, D	1.0
Crested drongo (*Dicrurus forficatus*)	Resting, Traveling	M	0.3
Crested coua (*Coua cristata*)	Resting, Traveling, Searching insects	M, D	0.3
Madagascar magpie-robin (*Copsychus albospecularis*)	Traveling	D	0.1
Total			100.0

Table S3. Upper-ranked models of generalized linear mixed model analysis for the effects of distance to mother trees, distance to conspecific fruiting trees, density of diaspores and canopy openness on seedling occurrence of *Astrotrichilia asterotricha* (< 2.0 of delta AIC).

Late December, 2015

Model	Intercept		Distance to mother tree		Distance to fruiting tree		Density of diaspores		Canopy openness		AIC	Akaike weight
Rank	Coefficient ± SE	P value	Coefficient ± SE	P value	Coefficient ± SE	P value	Coefficient ± SE	P value	Coefficient ± SE	P value		
1	-1.67 ± 0.39	< 0.0001	Not selected		Not selected		-0.040 ± 0.018	0.022	Not selected		417.3	0.32
2	-2.52 ± 0.93	0.0069	Not selected		Not selected		-0.042 ± 0.018	0.018	0.071 ± 0.071	0.32	418.3	0.20
3	-1.52 ± 0.55	0.0061	-0.0020 ± 0.0050	0.69	Not selected		-0.048 ± 0.026	0.066	Not selected		419.2	0.13
IOV in upper-ranked models			0.13		-		0.64		0.20			

Early January, 2016

Model	Intercept		Distance to mother tree		Distance to fruiting tree		Density of diaspores		Canopy openness		AIC	Akaike weight
Rank	Coefficient ± SE	P value	Coefficient ± SE	P value	Coefficient ± SE	P value	Coefficient ± SE	P value	Coefficient ± SE	P value		
1	-2.75 ± 0.73	0.0002	Not selected		Not selected		Not selected		0.099 ± 0.057	0.084	569.1	0.26
2	-1.56 ± 0.21	< 0.0001	Not selected		Not selected		Not selected		Not selected		570.1	0.16
3	-2.81 ± 0.73	0.0001	0.0018 ± 0.0029	0.54	Not selected		Not selected		0.10 ± 0.057	0.079	570.7	0.12
4	-2.71 ± 0.75	0.0003	Not selected		Not selected		-0.0038 ± 0.015	0.80	0.10 ± 0.057	0.081	571.0	0.10
5	-2.73 ± 0.74	0.0002	Not selected		-0.0018 ± 0.0098	0.85	Not selected		0.097 ± 0.057	0.089	571.1	0.098
IOV in upper-ranked models			0.12		0.098		0.10		0.58			

Late January, 2016

Model	Intercept		Distance to mother tree		Distance to fruiting tree		Density of diaspores		Canopy openness		AIC	Akaike weight
Rank	Coefficient ± SE	P value	Coefficient ± SE	P value	Coefficient ± SE	P value	Coefficient ± SE	P value	Coefficient ± SE	P value		
1	-3.09 ± 0.27	< 0.0001	0.0066 ± 0.0042	0.12	Not selected		Not selected		Not selected		257.9	0.25
2	-3.10 ± 0.28	< 0.0001	Not selected		0.020 ± 0.014	0.17	Not selected		Not selected		258.4	0.20
3	-3.64 ± 0.88	< 0.0001	0.011 ± 0.0078	0.16	Not selected		Not selected		0.030 ± 0.046	0.51	259.4	0.12
4	-2.66 ± 0.42	< 0.0001	Not selected		Not selected		-0.019 ± 0.025	0.45	Not selected		259.6	0.11
5	-3.23 ± 1.09	0.0032	0.0067 ± 0.0043	0.12	Not selected		Not selected		0.012 ± 0.087	0.89	259.9	0.092
IOV in upper-ranked models			0.46		0.20		0.22		0.092			

Early February, 2016

Model	Intercept		Distance to mother tree		Distance to fruiting tree		Density of diaspores		Canopy openness		AIC	Akaike weight
Rank	Coefficient ± SE	P value	Coefficient ± SE	P value	Coefficient ± SE	P value	Coefficient ± SE	P value	Coefficient ± SE	P value		
1	-2.66 ± 0.49	< 0.0001	Not selected		Not selected		-0.056 ± 0.029	0.053	Not selected		194.6	0.30
2	-3.68 ± 0.39	< 0.0001	Not selected		0.028 ± 0.017	0.099	Not selected		Not selected		195.6	0.17
3	-2.32 ± 0.74	0.0016	-0.046 ± 0.0076	0.55	Not selected		-0.073 ± 0.040	0.067	Not selected		196.2	0.13
4	-3.05 ± 1.39	0.028	Not selected		Not selected		-0.056 ± 0.029	0.05	0.032 ± 0.11	0.77	196.5	0.11
IOV in upper-ranked models			0.13		0.17		0.55		0.11			

Late February, 2016

Model	Intercept		Distance to mother tree		Distance to fruiting tree		Density of diaspores		Canopy openness		AIC	Akaike weight
Rank	Coefficient ± SE	P value	Coefficient ± SE	P value	Coefficient ± SE	P value	Coefficient ± SE	P value	Coefficient ± SE	P value		
1	-8.98 ± 3.21	0.0052	-0.016 ± 0.012	0.16	Not selected		-0.16 ± 0.058	0.0065	0.51 ± 0.23	0.029	101.9	0.32
2	-9.16 ± 2.92	0.0017	Not selected		Not selected		-0.10 ± 0.045	0.027	0.44 ± 0.20	0.032	102.1	0.29
3	-10.73 ± 2.89	0.0002	Not selected		-0.052 ± 0.025	0.036	Not selected		0.42 ± 0.20	0.031	103.0	0.19
IOV in upper-ranked models			0.32		0.19		0.61		0.79			

Early March, 2016

Model	Intercept		Distance to mother tree		Distance to fruiting tree		Density of diaspores		Canopy openness		AIC	Akaike weight
Rank	Coefficient ± SE	P value	Coefficient ± SE	P value	Coefficient ± SE	P value	Coefficient ± SE	P value	Coefficient ± SE	P value		
1	-9.81 ± 3.30	0.0029	Not selected		Not selected		-0.11 ± 0.051	0.029	0.49 ± 0.23	0.032	88.6	0.32
2	-11.51 ± 3.26	0.0004	Not selected		0.060 ± 0.027	0.028	Not selected		0.47 ± 0.22	0.032	89.1	0.25
3	-9.68 ± 3.57	0.0066	-0.013 ± 0.012	0.27	Not selected		-0.16 ± 0.066	0.014	0.55 ± 0.26	0.032	89.3	0.23
IOV in upper-ranked models			0.23		0.25		0.55		0.80			

Late March, 2016

Model	Intercept		Distance to mother tree		Distance to fruiting tree		Density of diaspores		Canopy openness		AIC	Akaike weight
Rank	Coefficient ± SE	P value	Coefficient ± SE	P value	Coefficient ± SE	P value	Coefficient ± SE	P value	Coefficient ± SE	P value		
1	-9.91 ± 3.44	0.0039	Not selected		Not selected		-0.15 ± 0.060	0.013	0.53 ± 0.24	0.029	79.4	0.36
2	-11.88 ± 3.37	0.0004	Not selected		0.077 ± 0.030	0.0091	Not selected		0.49 ± 0.22	0.028	80.1	0.26
3	-9.82 ± 3.75	0.0088	-0.013 ± 0.012	0.27	Not selected		-0.20 ± 0.074	0.0070	0.59 ± 0.27	0.031	80.1	0.26
IOV in upper-ranked models			0.26		0.26		0.62		0.88			

Early April, 2016

Model	Intercept		Distance to mother tree		Distance to fruiting tree		Density of diaspores		Canopy openness		AIC	Akaike weight
Rank	Coefficient ± SE	P value	Coefficient ± SE	P value	Coefficient ± SE	P value	Coefficient ± SE	P value	Coefficient ± SE	P value		
1	-8.75 ± 3.62	0.016	Not selected		Not selected		-0.18 ± 0.075	0.015	0.43 ± 0.25	0.086	70.1	0.32
2	-8.62 ± 3.92	0.028	-0.012 ± 0.012	0.30	Not selected		-0.23 ± 0.085	0.0074	0.49 ± 0.28	0.082	70.9	0.21
3	-11.33 ± 3.44	0.0010	Not selected		0.087 ± 0.033	0.0088	Not selected		0.41 ± 0.23	0.077	71.4	0.16
4	-3.40 ± 1.15	0.0032	Not selected		Not selected		-0.17 ± 0.073	0.019	Not selected		71.6	0.15
IOV in upper-ranked models			0.21		0.16		0.67		0.69			

Late April, 2016

Model	Intercept		Distance to mother tree		Distance to fruiting tree		Density of diaspores		Canopy openness		AIC	Akaike weight
Rank	Coefficient ± SE	P value	Coefficient ± SE	P value	Coefficient ± SE	P value	Coefficient ± SE	P value	Coefficient ± SE	P value		
1	-8.75 ± 3.62	0.016	Not selected		Not selected		-0.18 ± 0.075	0.015	0.43 ± 0.25	0.086	70.1	0.32
2	-8.62 ± 3.92	0.028	-0.012 ± 0.012	0.30	Not selected		-0.23 ± 0.085	0.0074	0.49 ± 0.28	0.082	70.9	0.21
3	-11.33 ± 3.44	0.0010	Not selected		0.087 ± 0.033	0.0088	Not selected		0.41 ± 0.23	0.077	71.4	0.16
4	-3.40 ± 1.15	0.0032	Not selected		Not selected		-0.17 ± 0.073	0.019	Not selected		71.6	0.15
IOV in upper-ranked models			0.21		0.16		0.67		0.69			

Early May, 2016

Model	Intercept		Distance to mother tree		Distance to fruiting tree		Density of diaspores		Canopy openness		AIC	Akaike weight
Rank	Coefficient ± SE	P value	Coefficient ± SE	P value	Coefficient ± SE	P value	Coefficient ± SE	P value	Coefficient ± SE	P value		
1	-13.00 ± 3.55	0.0003	Not selected		0.13 ± 0.047	0.0049	Not selected		0.46 ± 0.22	0.036	51.7	-

Latter May, 2016

Model	Intercept		Distance to mother tree		Distance to fruiting tree		Density of diaspores		Canopy openness		AIC	Akaike weight
Rank	Coefficient ± SE	P value	Coefficient ± SE	P value	Coefficient ± SE	P value	Coefficient ± SE	P value	Coefficient ± SE	P value		
1	-13.00 ± 3.55	0.0003	Not selected		0.13 ± 0.047	0.0049	Not selected		0.46 ± 0.22	0.036	51.7	-

Late November, 2016

Model	Intercept		Distance to mother tree		Distance to fruiting tree		Density of diaspores		Canopy openness		AIC	Akaike weight
Rank	Coefficient ± SE	P value	Coefficient ± SE	P value	Coefficient ± SE	P value	Coefficient ± SE	P value	Coefficient ± SE	P value		
1	-20.79 ± 6.98	0.0029	Not selected		0.21 ± 0.11	0.046	Not selected		0.82 ± 0.35	0.02	30.3	-

Late February, 2017

Model	Intercept		Distance to mother tree		Distance to fruiting tree		Density of diaspores		Canopy openness		AIC	Akaike weight
Rank	Coefficient ± SE	P value	Coefficient ± SE	P value	Coefficient ± SE	P value	Coefficient ± SE	P value	Coefficient ± SE	P value		
1	-23.62 ± 9.09	0.0094	Not selected		0.15 ± 0.077	0.058	Not selected		1.09 ± 0.54	0.045	24.1	-

Table S4. Upper-ranked models of generalized linear mixed model analysis for the effects of distance to mother trees, distance to conspecific fruiting trees, density of diaspores and canopy openness on seedling occurrence of *Abrahamia deflexa* (< 2.0 of delta AIC).

Early February, 2016

Model	Intercept		Distance to mother tree		Distance to fruiting tree		Density of diaspores		Canopy openness		AIC	Akaike weight
Rank	Coefficient ± SE	P value	Coefficient ± SE	P value	Coefficient ± SE	P value	Coefficient ± SE	P value	Coefficient ± SE	P value		
1	-1.31 ± 1.19	0.27	Not selected		Not selected		-0.10 ± 0.016	< 0.0001	0.13 ± 0.086	0.14	501.7	0.27
2	-0.11 ± 0.46	0.81	0.0062 ± 0.0042	0.14	Not selected		-0.080 ± 0.023	0.0004	Not selected		501.8	0.25
3	0.39 ± 0.31	0.22	Not selected		Not selected		-0.10 ± 0.015	< 0.0001	Not selected		502.0	0.23
4	-1.55 ± 1.21	0.2	0.0054 ± 0.0042	0.2	Not selected		-0.079 ± 0.023	0.0006	0.11 ± 0.087	0.19	502.0	0.22
IOV in upper-ranked models			0.47		-		0.96		0.49			

Late February, 2016

Model	Intercept		Distance to mother tree		Distance to fruiting tree		Density of diaspores		Canopy openness		AIC	Akaike weight
Rank	Coefficient ± SE	P value	Coefficient ± SE	P value	Coefficient ± SE	P value	Coefficient ± SE	P value	Coefficient ± SE	P value		
1	-2.35 ± 1.31	0.072	0.0061 ± 0.0043	0.16	Not selected		-0.087 ± 0.024	0.0003	0.15 ± 0.094	0.11	434.1	0.30
2	-2.07 ± 1.28	0.11	Not selected		Not selected		-0.11 ± 0.017	< 0.0001	0.17 ± 0.093	0.071	434.1	0.30
3	-0.41 ± 0.45	0.37	0.0069 ± 0.0042	0.098	Not selected		-0.089 ± 0.024	0.0002	Not selected		434.9	0.20
4	0.15 ± 0.29	0.60	Not selected		Not selected		-0.12 ± 0.017	< 0.0001	Not selected		435.6	0.14
IOV in upper-ranked models			0.51		-		0.94		0.60			

Early March, 2016

Model	Intercept		Distance to mother tree		Distance to fruiting tree		Density of diaspores		Canopy openness		AIC	Akaike weight
Rank	Coefficient ± SE	P value	Coefficient ± SE	P value	Coefficient ± SE	P value	Coefficient ± SE	P value	Coefficient ± SE	P value		
1	-1.92 ± 1.29	0.14	Not selected		Not selected		-0.12 ± 0.018	< 0.0001	0.15 ± 0.094	0.11	411.2	0.29
2	-2.15 ± 1.32	0.10	0.0055 ± 0.0043	0.20	Not selected		-0.096 ± 0.025	0.0001	0.14 ± 0.095	0.15	411.6	0.24
3	-0.42 ± 0.46	0.36	0.0063 ± 0.0042	0.13	Not selected		-0.098 ± 0.025	< 0.0001	Not selected		411.8	0.22
4	0.10 ± 0.29	0.73	Not selected		Not selected		-0.12 ± 0.017	< 0.0001	Not selected		412.0	0.19
IOV in upper-ranked models			0.46		-		0.94		0.53			

Late March, 2016

Model	Intercept		Distance to mother tree		Distance to fruiting tree		Density of diaspores		Canopy openness		AIC	Akaike weight
Rank	Coefficient ± SE	P value	Coefficient ± SE	P value	Coefficient ± SE	P value	Coefficient ± SE	P value	Coefficient ± SE	P value		
1	-0.49 ± 0.46	0.29	0.0071 ± 0.0042	0.095	Not selected		-0.098 ± 0.025	< 0.0001	Not selected		400.8	0.28
2	-2.05 ± 1.32	0.12	0.0064 ± 0.0043	0.14	Not selected		-0.096 ± 0.025	0.0001	0.12 ± 0.096	0.20	401.0	0.24
3	-1.77 ± 1.30	0.17	Not selected		Not selected		-0.12 ± 0.018	< 0.0001	0.14 ± 0.094	0.14	401.2	0.22
4	0.089 ± 0.30	0.76	Not selected		Not selected		-0.13 ± 0.018	< 0.0001	Not selected		401.6	0.18
IOV in upper-ranked models			0.52		-		0.92		0.46			

Early April, 2016

Model	Intercept		Distance to mother tree		Distance to fruiting tree		Density of diaspores		Canopy openness		AIC	Akaike weight
Rank	Coefficient ± SE	P value	Coefficient ± SE	P value	Coefficient ± SE	P value	Coefficient ± SE	P value	Coefficient ± SE	P value		
1	-0.56 ± 0.47	0.23	0.0072 ± 0.0043	0.093	Not selected		-0.096 ± 0.025	0.0001	Not selected		394.6	0.27
2	-2.12 ± 1.33	0.11	0.0065 ± 0.0043	0.14	Not selected		-0.094 ± 0.025	0.0002	0.12 ± 0.096	0.20	394.9	0.23
3	-1.83 ± 1.30	0.16	Not selected		Not selected		-0.12 ± 0.018	< 0.0001	0.14 ± 0.095	0.14	395.1	0.21
4	0.028 ± 0.29	0.92	Not selected		Not selected		-0.13 ± 0.018	< 0.0001	Not selected		395.5	0.17
IOV in upper-ranked models			0.50		-		0.88		0.44			

Late April, 2016

Model	Intercept		Distance to mother tree		Distance to fruiting tree		Density of diaspores		Canopy openness		AIC	Akaike weight
Rank	Coefficient ± SE	P value	Coefficient ± SE	P value	Coefficient ± SE	P value	Coefficient ± SE	P value	Coefficient ± SE	P value		
1	-0.56 ± 0.47	0.23	0.0065 ± 0.0043	0.13	Not selected		-0.095 ± 0.025	0.0002	Not selected		397.7	0.28
2	-0.025 ± 0.30	0.93	Not selected		Not selected		-0.12 ± 0.018	< 0.0001	Not selected		398.0	0.23
3	-1.63 ± 1.28	0.21	Not selected		Not selected		-0.12 ± 0.018	< 0.0001	0.12 ± 0.094	0.20	398.2	0.21
4	-1.90 ± 1.31	0.14	0.0059 ± 0.0044	0.17	Not selected		-0.093 ± 0.025	0.0002	0.11 ± 0.095	0.27	398.4	0.19
IOV in upper-ranked models			0.47		-		0.91		0.40			

Early May, 2016

Model	Intercept		Distance to mother tree		Distance to fruiting tree		Density of diaspores		Canopy openness		AIC	Akaike weight
Rank	Coefficient ± SE	P value	Coefficient ± SE	P value	Coefficient ± SE	P value	Coefficient ± SE	P value	Coefficient ± SE	P value		
1	-0.16 ± 0.29	0.58	Not selected		Not selected		-0.12 ± 0.018	< 0.0001	Not selected		390.0	0.32
2	-0.58 ± 0.46	0.21	0.0052 ± 0.0043	0.23	Not selected		-0.094 ± 0.025	0.0002	Not selected		390.5	0.24
3	-1.36 ± 1.27	0.29	Not selected		Not selected		-0.11 ± 0.018	< 0.0001	0.091 ± 0.093	0.33	391.0	0.19
4	-1.58 ± 1.29	0.22	0.0047 ± 0.0044	0.28	Not selected		-0.093 ± 0.026	0.0003	0.078 ± 0.093	0.40	391.8	0.13
IOV in upper-ranked models			0.37		-		0.89		0.32			

Late May, 2016

Model	Intercept		Distance to mother tree		Distance to fruiting tree		Density of diaspores		Canopy openness		AIC	Akaike weight
Rank	Coefficient ± SE	P value	Coefficient ± SE	P value	Coefficient ± SE	P value	Coefficient ± SE	P value	Coefficient ± SE	P value		
1	-0.15 ± 0.30	0.61	Not selected		Not selected		-0.12 ± 0.018	< 0.0001	Not selected		384.4	0.33
2	-0.58 ± 0.47	0.22	0.0052 ± 0.0043	0.23	Not selected		-0.097 ± 0.026	0.0001	Not selected		385.0	0.25
3	-1.28 ± 1.28	0.32	Not selected		Not selected		-0.12 ± 0.018	< 0.0001	0.085 ± 0.094	0.36	385.5	0.19
4	-1.49 ± 1.30	0.25	0.0049 ± 0.0044	0.28	Not selected		-0.096 ± 0.026	0.0001	0.072 ± 0.094	0.45	386.3	0.13
IOV in upper-ranked models			0.38		-		0.90		0.32			

Late November, 2016

Model	Intercept		Distance to mother tree		Distance to fruiting tree		Density of diaspores		Canopy openness		AIC	Akaike weight
Rank	Coefficient ± SE	P value	Coefficient ± SE	P value	Coefficient ± SE	P value	Coefficient ± SE	P value	Coefficient ± SE	P value		
1	-1.61 ± 0.63	0.011	0.011 ± 0.0053	0.045	Not selected		-0.12 ± 0.038	0.0016	Not selected		227.0	0.31
2	-3.78 ± 0.43	< 0.0001	Not selected		0.078 ± 0.012	< 0.0001	Not selected		Not selected		227.1	0.30
3	-2.22 ± 1.70	0.19	0.010 ± 0.0054	0.051	Not selected		-0.12 ± 0.038	0.0019	0.047 ± 0.12	0.70	228.8	0.12
IOV in upper-ranked models			0.43		0.30		0.43		0.12			

Late February, 2017

Model	Intercept		Distance to mother tree		Distance to fruiting tree		Density of diaspores		Canopy openness		AIC	Akaike weight
Rank	Coefficient ± SE	P value	Coefficient ± SE	P value	Coefficient ± SE	P value	Coefficient ± SE	P value	Coefficient ± SE	P value		
1	-4.00 ± 0.53	< 0.0001	Not selected		0.073 ± 0.013	< 0.0001	Not selected		Not selected		208.9	0.31
2	-2.09 ± 0.75	0.0054	0.011 ± 0.0058	0.051	Not selected		-0.10 ± 0.040	0.0091	Not selected		209.1	0.28
3	-3.81 ± 1.65	0.021	Not selected		0.074 ± 0.013	< 0.0001	Not selected		-0.015 ± 0.13	0.90	210.9	0.11
IOV in upper-ranked models			0.28		0.43		0.28		0.11			

Early March, 2018

Model	Intercept		Distance to mother tree		Distance to fruiting tree		Density of diaspores		Canopy openness		AIC	Akaike weight
Rank	Coefficient ± SE	P value	Coefficient ± SE	P value	Coefficient ± SE	P value	Coefficient ± SE	P value	Coefficient ± SE	P value		
1	-4.69 ± 0.57	< 0.0001	0.029 ± 0.0059	< 0.0001	Not selected		Not selected		Not selected		125.7	0.35
2	-4.94 ± 0.65	< 0.0001	Not selected		0.085 ± 0.019	< 0.0001	Not selected		Not selected		126.6	0.22
3	-4.14 ± 1.26	0.0010	0.025 ± 0.0099	0.013	Not selected		-0.034 ± 0.072	0.64	Not selected		127.4	0.14
4	-3.94 ± 2.37	0.096	0.029 ± 0.0060	< 0.0001	Not selected		Not selected		-0.058 ± 0.18	0.75	127.6	0.13
IOV in upper-ranked models			0.63		0.22		0.14		0.13			
